# High and Low Haemoglobin Levels in Early Pregnancy Are Associated to a Higher Risk of Miscarriage: A Population-Based Cohort Study

**DOI:** 10.3390/nu13051578

**Published:** 2021-05-08

**Authors:** Andrés Díaz-López, Blanca Ribot, Josep Basora, Victoria Arija

**Affiliations:** 1 Serra Hunter Fellow, Universitat Rovira i Virgili (URV), 43201 Reus, Spain; andres.diaz@iispv.cat; 2 Nutrition and Mental Health Research Group (NUTRISAM), Rovira i Virgili University (URV), 43201 Reus, Spain; blanca.ribot@uvic.cat; 3 Institut d’Investigació Sanitària Pere Virgili (IISPV), 43003 Reus, Spain; 4 CIBER of Pathophysiology of Obesity and Nutrition (CIBEROBN), Instituto de Salud Carlos III, 28029 Madrid, Spain; 5 Research Group on Methodology, Methods, Models and Outcomes of Health and Social Sciences (M3O), Faculty of Health Sciences and Welfare, Centre for Health and Social Care Research (CESS), University of Vic-Central University of Catalonia (UVIC-UCC), 08500 Vic, Spain; 6 Unitat de Suport a la Recerca Tarragona-Reus, Fundació Institut Universitari per a la Recerca a l’Atenció Primària de Salut Jordi Gol i Gurina (IDIAPJGol), 43202 Reus, Spain; jbasora@idiapjgol.org

**Keywords:** miscarriage, pregnancy loss, anaemia, high Hb concentration

## Abstract

To evaluate whether women with anaemia or high haemoglobin (Hb) in early pregnancy would be at higher risk of miscarriage, this population-based cohort study involved 9453 women whose pregnancies were monitored at primary care centres between 2007 and 2012. The computerised clinical histories were used to collect: Hb measurements (up to 14 weeks of gestation), miscarriage before or by 24 weeks of gestation, and other maternal characteristics. The relation between anaemia (Hb < 110 g/L), normal Hb (110–140 g/L, reference), and high Hb concentrations (≥140 g/L) with miscarriage were expressed as adjusted OR with 95%CI. Restricted cubic spline models were applied to evaluate the dose-response relationships. A total of 520 (5.5%) women were recorded as having a miscarriage. The rate of miscarriage in anaemia, normal Hb, and high Hb concentrations was 8.4%, 5.1%, and 10.2%, respectively. Compared with women with normal Hb at the first trimester, the multivariable-adjusted OR for miscarriage was 2.11 (95%CI, 1.38–3.21) for women with anaemia and 1.83 (95%CI, 1.29–2.58) for women with high Hb. Hb concentrations showed a U-shaped association with miscarriage, with the lowest incidence among women with Hb of 120–130 g/L. These data highlight the importance of considering anaemia and high Hb levels in early pregnancy as harmful indicators for miscarriage.

## 1. Introduction

Miscarriage, 85% of which may happen during the first trimester, is one of the most common adverse pregnancy outcomes [1,2]. Although the cause of most miscarriages remains unknown, they presumably result from a complex interplay of non-modifiable and modifiable risk factors [3,4]. It has been suggested that 50% of miscarriages, especially those of the first trimester, are attributed to chromosomal abnormalities [5,6]; however, other maternal factors may also play a role. Therefore, it is essential, in order to guide future public health policies, to identify risk factors prior to and during pregnancy. In fact, scientific research supports that more than a quarter of miscarriages would be preventable by reducing the associated modifiable risk factors [7,8,9,10]. Well-known risk factors include advanced age, pre-existing comorbidities (obesity, diabetes, or hypertension), previous miscarriages, smoking, or inappropriate nutritional status [3,9,11].

Anaemia, event preventable, is the most prominent haematological abnormality during gestation and it is a global health problem affecting nearly half of all pregnant women [12]. Anaemia has been linked to a higher risk of adverse consequences, including maternal mortality, stillbirth, preterm births (PTB), low birth weight (LBW), small-for-gestational-age (SGA), as well as other gestational complications [13,14,15]. Moreover, previous studies have also reported a U-shaped curve of increased risk by maternal Hb for some of these adverse outcomes [14,16,17], but relations considerably differed by the time at which Hb level was assessed. Furthermore, high Hb concentration, particularly in early pregnancy, has received much less attention than anaemia because it is often perceived as a sign of adequate iron nutrition. In this context, existing evidence focusing on anaemia or high Hb concentrations during early pregnancy and miscarriage risk is scarce and the results are inconclusive [18,19,20]; therefore, it warrants further investigation. Moreover, to date, studies on this matter conducted in the Mediterranean and southern European countries with different dietary and lifestyle habits or non-modifiable (genetics) factors are still lacking.

Recognizing the lack of available evidence, the aim of this study was to investigate the impact of anaemia and high Hb concentrations during early pregnancy (up to 14 weeks of gestation) on miscarriage risk in pregnant Spanish women on the eastern coast of the Mediterranean.

## 2. Materials and Methods

### 2.1. Study Design and Participants

The current study is a longitudinal, population-based cohort study involving pregnant women with singleton pregnancies who undertook monitoring and control of the pregnancy exclusively in the Sexual and Reproductive Health Care Service (ASSIR) of the Tarragona and Reus, Spain, between 2007 and 2012. The ASSIR of the Catalan Health Institute (ICS) is a support service provided by obstetricians, gynaecologists, and midwives whose aim is to promote and coordinate comprehensive sexual and reproductive healthcare for pregnant women attending primary health care. The ICS began to computerise clinical records in 2006 by assigning one health identifier to each patient for health care. Furthermore, pregnant women are assigned a unique identification number for each pregnancy so it can be monitored individually. The ASSIR records include data on pregnancy, delivery, and the postpartum monitoring collected according to the “pregnancy monitoring protocol in force in Catalonia” (Protocol on pregnancy monitoring in Catalonia) [21]. Briefly, in ASSIR, frequencies of visits depend on the individual woman’s needs and are based on her associated risk factors. Pregnancy assistance begins at the first recruitment visit, near the end of the first trimester, approximately between weeks 8 and 10. After the first visit, a minimum of 8 monitoring visits during pregnancy is recommended for women with uncomplicated pregnancies with the following periodicity: at 11 to 13 weeks, 16 to 17 weeks, 20 to 21 weeks, 25 to 26 weeks, 29 to 30 weeks, 34 to 36 weeks, 38 to 40 weeks, and, finally, at 41 weeks of gestation. Despite this, women with problems are seen more frequently depending on the nature of the problems. In total, most women have at least 3 transvaginal or abdominal ultrasound examinations during pregnancy: at 11.2–13.6 weeks, 19–22 weeks, and 34–36 weeks, at each corresponding visit.

A total of 13,185 pregnant women were recruited by the ASSIR in Tarragona and Reus and followed until the post-partum period. Of these, 11,259 women underwent blood analyses, and 9,488 women did so during the first trimester [22]. For the current analysis, our main objective was to determine the effect of first trimester Hb levels on miscarriage incidence before or by 24 weeks of gestation. Therefore, women who experienced a foetal death of a clinically recognized pregnancy after 24 weeks of gestation were excluded from the main analysis (*n* = 35). Overall, a total of 9453 pregnant women with data available on Hb measurements up to 14 weeks of gestation were included in the final analysis.

It is opportune to mention that the ICS recommends that all pregnant women take a daily dose of around 40 mg of iron from the start of the second trimester unless they have hemochromatosis. For anaemic pregnant women, this is increased to 80 mg of iron 1 or 2 times a day depending on the severity of iron deficiency. Consequently, we considered that pregnant women were not receiving any treatment at the time of blood collection during the first trimester.

### 2.2. Data Collection

The ASSIR administrative database or electronic medical record of each pregnancy were used to collect demographic data (age of the mother at conception) and gynaecological-obstetric and prenatal history (date of the last menstruation, date of pregnancy losses, date of delivery, weight and height in the first trimester, tobacco use during pregnancy (yes/no), parity and number of previous pregnancies, and number of previous abortions). However, there were no data on the extent to which the women complied with the recommendations regarding the use of iron supplements.

Maternal age (years) was classified as <20, 20–24, 25–29, 30–34, 35–39, or ≥40 years. Maternal body mass index (BMI, Kg/m^2^) was calculated as weight (kg) divided by height (m) squared from height and weight recorded by midwives in the first trimester. After, BMI was categorized as underweight (BMI < 18.5 Kg/m^2^), normal weight (BMI 18.5–24.9 Kg/m^2^), overweight, or obese (BMI ≥ 25 Kg/m^2^). Parity was categorised as nulliparous (no previous live birth) or parous (one or more live births). The number of previous abortions was categorised as 0, 1, or ≥2. Gestational age (weeks) was estimated from the date of the first day of the last menstrual period provided by women to the date of miscarriage register or end of follow-up (delivery).

Furthermore, data about the maternal Hb up to 14 weeks of gestation was also obtained from ASSIR database. Fasting blood samples were collected from pregnant women by the primary care nurses (between 8–9 a.m.) for routine blood testing at each trimester, including Hb assessment. Each pregnant woman had between 1 and 7 Hb (mean = 1.2) measures taken between gestational weeks 0–14. In our study, data on Hb referred to the first value available based on a blood test at the beginning of the pregnancy or test requested at the first prenatal visit. The vast majority (80%) of Hb determinations were done within 9 and 11 weeks of gestation. In all cases, Hb measurements were available prior to the outcome.

According to the definition of anaemia during pregnancy (Hb < 110 g/L, corresponding to below the 5th percentile) and the 95th percentile of the Hb level in our study (140 g/L), women were classified into 3 groups: anaemia, Hb concentration <110 g/L; normal Hb concentration; Hb concentration in the range of 110–140 g/L (reference category); and high Hb concentration, Hb ≥ 140 g/L.

### 2.3. Outcome

The main pregnancy outcome for this study was miscarriage until 24 weeks of gestation. All miscarriage cases were diagnosed and confirmed by obstetricians and/or gynaecologists. Pregnancy was initially documented via a positive urine or blood pregnancy test. In our study, miscarriage incidence was defined as the involuntary termination of a pregnancy; according to the transvaginal ultrasound findings, which were usually accompanied by low serum β-human chorionic gonadotrophin levels, miscarriage was considered as a pre-embryonic loss with an empty gestational sac or a yolk sac, or embryonic loss with a detectable embryo, or foetal loss.

### 2.4. Statistical Analyses

Statistical analyses were performed using STATA, version 15.0 (StataCorp LP, College Station, TX, USA). Descriptive data are presented as mean±SD or number (%). Comparisons between groups were carried out using independent-sample t-test, ANOVA, or chi-square tests, as appropriate.

Using univariable and multivariable logistic regression analyses, we estimated the odds ratio (OR) and 95% confidence interval (CI) of miscarriage by maternal Hb (<110, 110–140 (reference), ≥140 g/L). Model was adjusted for maternal age (<20, 20–24, 25–29 (reference), 30–34, 35–39, or ≥40 years), BMI (<18.5, 18.5–24.9 (reference), ≥25 Kg/m^2^), parity (nulliparous (reference), parous), smoking (no, yes), and prior miscarriage (0 (reference), 1, or ≥2). A restricted cubic spline analysis with 3 knots and Hb levels of 110 g/L set as the referent was applied to evaluate the dose-response relationships. The three knot locations for Hb levels were 115, 127, and 137 g/L.

Sensitivity analyses assessing the consistency of our results under various scenarios were conducted. Firstly, we analysed separately miscarriage at two different stages of pregnancy: early miscarriage (up to 14 weeks of gestation) and second-trimester miscarriage (from 15–24 weeks of gestation). Secondly, a sensitivity analysis was also conducted by excluding miscarriages documented within 11 and 14 weeks of gestation with a period of fewer than 2 weeks from the date of the Hb tests. The purpose of the latter analysis was to avoid reverse causality. During 11 to 14 weeks of gestation, occasionally, there are no noticeable symptoms (e.g., spotting or bleeding) and therefore could be a silent miscarriage with a delayed record. Thirdly, the analysis was repeated including all pregnancy loss documented up to and beyond 24 weeks of gestation.

Finally, motivated by the possibility that the associations observed were due to confounding factors, we investigated whether the effect of Hb on miscarriage differed by factors strongly related to pregnancy loss, such as pregnancy BMI (<25 vs. ≥25 Kg/m^2^) and maternal age (≤29 vs. >29 years), using stratified analyses. Likelihood ratio tests were used to test the interaction terms by comparing the model with and without the product term. Statistical significance was set at *p* < 0.05.

## 3. Results

Among 9453 eligible women (age 29.9 ± 5.5 years), the mean maternal Hb level in the first trimester was 126.3 ± 9.0 g/L. There was no statistically significant difference between women included and excluded from study analysis for characteristics at baseline (age at conception, BMI in the first trimester, smoking habit, previous births, or parity; *p* > 0.10). The total incidence of miscarriage by 24 weeks of gestation was 5.5% (4.6% before the end of the 14th week and 1.0% between 15–24 weeks of gestation).

Compared to women with normally progressing pregnancies, women with miscarriage were older (29.9 ± 5.5 vs. 31.5 ± 6.0 years, *p* < 0.001), had higher BMI (24.7 ± 4.5 vs. 25.3 ± 4.6 Kg/m^2^, *p* = 0.006), and were more often nulliparous (43.3 vs. 48.6%, *p* = 0.02).

Table 1 shows the general and obstetric characteristics of pregnant women by groups of Hb concentrations up to 14 weeks of gestation. The rate of miscarriage in anaemia, normal Hb, and high Hb concentration was 8.4%, 5.1%, and 10.2%, respectively. Compared to women with normal Hb concentrations, women with anaemia smoked less and were more likely to have a higher number of previous births, pregnancies, and abortions; women with high Hb concentrations were also older, had higher body weight and BMI, were less likely to be a smoker, and had fewer deliveries.

The OR of having a miscarriage was calculated taking as reference those women with normal Hb concentrations (Table 2). After adjusting for all potential confounders, the risk of miscarriage was significantly increased among pregnant women with anaemia (OR, 2.11; 95%CI, 1.38 to 3.21) or high Hb concentrations (OR, 1.83; 95%CI, 1.29 to 2.58). In accordance with previous studies, the groups of 30–34, 35–39, and ≥40 years were associated with miscarriage. No association was found between BMI, smoking habit, or previous miscarriage history and miscarriage in the fully adjusted model.

**Figure 1:**
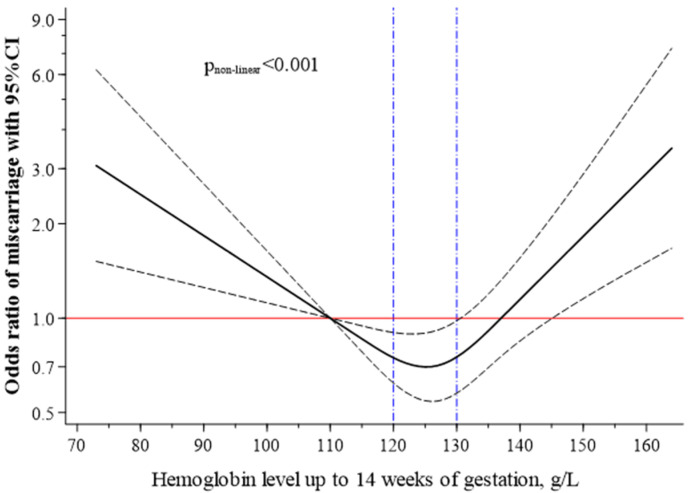
Association between Hb levels up to 14 weeks of gestation as a continuous variable and the risks of miscarriage. Restricted cubic spline analysis with 3 knots and haemoglobin levels 110 g/L set as the referent. The plot shows adjusted ORs (solid line) with 95%CI (dashed lines). Logistic regression model adjusted for the same characteristics or covariates as Table 2.

To account for non-linear associations between maternal Hb and miscarriage, we used restricted cubic spline analysis with 3 knots using the WHO cut-off for anaemia (Hb 110 g/L) as a reference value. Figure 1 shows that the maternal Hb-associated risk of miscarriage had a U-shaped pattern, increasing significantly at Hb levels below 110 g/dL and above 140 g/dL. The optimal maternal Hb concentrations, with the lowest miscarriage incidence, were between 120 and 130 g/L.

Our main results on Hb levels and miscarriage risk showed no substantial differences when subjected to several sensitivity analyses (Table 3). Results were similar when analyses were restricted to early miscarriage, second-trimester miscarriage, or when loss of pregnancy after 24 weeks of gestation were considered. Similarly, following the exclusion of miscarriages documented with a period of fewer than 2 weeks from the date of the Hb tests, the results did not appreciably differ from those for the entire sample (Table 3).

Finally, we conducted stratified analyses to evaluate whether the maternal Hb-miscarriage association varies according to BMI (< 25 vs. ≥25 Kg/m^2^) and maternal age (≤29 vs. >29 years), and the linkage observed for low and high Hb concentration with an elevated risk of miscarriage was persistent across subgroups (Table 4). We found no significant interaction.

## 4. Discussion

To the best of our knowledge, this is the first population-based cohort study to show an association of anaemia and high Hb concentrations in early pregnancy (up to 14 weeks of gestation) with an increased risk of miscarriage in women from the western coast of the Mediterranean. These associations were independent of well-established risk factors. Our findings indicate an advance in the understanding of the risk factors of miscarriages and support the relevance of monitoring Hb concentrations in early pregnancy and providing appropriate intervention.

To date, there are numerous systematic reviews and meta-analyses of prospective cohorts that relate maternal anaemia during the first trimester of pregnancy with increased risks for certain adverse pregnancy outcomes [16,23], but evidence related to high Hb concentrations is more limited [14,15,17,24], and none of them analysed their effect on the risk of miscarriages. There are only three studies that have assessed the impact of maternal Hb concentrations during early pregnancy on subsequent miscarriage, and they provided conflicting results [18,19,20].

Unlike our results, at least in part, recently, a large case-control study conducted in Finland with 22,271 pregnant women [18] and another prospective study conducted in 817 middle-aged Sri Lankan women [20] found no additional risk of miscarriage when Hb < 100 g/L and Hb < 110 g/L during the first trimester, respectively. On the contrary, a large-scale population-based cohort study including 3,971,428 Chinese women reported an increased risk of miscarriage for women with severe anaemia prior to pregnancy (Hb < 70 g/L) [19]. It is noteworthy that in our study population, few women (<0.5%) had Hb concentrations of <80 g/L, which made comparisons difficult at the low extreme of the Hb.

The lack of agreement between the few studies that assess this association may be due to the fact that the populations come from countries with very different lifestyles, as well as due to the different designs and methodologies used in the studies. This indicates the need to increase prospective studies in which the causal factor is prior to the effect and in which the relationship between Hb levels and miscarriage is adjusted for other confounding factors. Furthermore, we must consider the bias that can occur in cross-sectional studies when interpreting the results. The transport of iron to the foetus is unidirectional through the placenta. It is possible that a non-evolutionary pregnancy causes an iron accumulation in the maternal plasma, increasing both iron and Hb concentrations; therefore, reverse causality cannot be excluded [25]. Thus, in studies that have not carefully reviewed the distance in time since the determination of Hb and the miscarriage, there would be a false decrease in the effect that anaemia may have on spontaneous abortion. Likewise, the estimate of the effect of iron excess would also be falsely increased. In our study, we avoided this confounder by using Hb levels prior to miscarriage, and our sensitivity analyses supported the robustness of the findings. The stronger associations in second-trimester miscarriage also argue against reverse causation.

Although the physiopathology underlying the anaemia-miscarriage relationship remains unclear, it appears that iron deficiency could play an important role by its relationship with hypoxia, oxidative stress, and increased infections [26]. Hypoxia caused by iron deficiency may initiate a response that results in increased cortisol production by the foetus, which is implicated in preterm labour [26]. Furthermore, iron deficiency increases oxidative stress since iron is part of the enzymatic antioxidant mechanism. This oxidative stress can damage the development of the placenta even in early pregnancy [27]. It should be noted that iron homeostasis changes once pregnant as well as the iron requirements. During pregnancy, iron requirements are increased and hepcidin levels are lower, favouring a greater absorption of dietary iron [25,28,29]. However, contrary to expectations and for reasons still unknown, women with miscarriage have a higher hepcidin concentration compared with normal first-trimester pregnancies regardless of their Hb levels [25].

Furthermore, our findings show that raised Hb levels in early pregnancy can be as harmful as iron deficiency anaemia in terms of miscarriage and that the rate of high Hb levels during early pregnancy could be even more frequent than anaemia (6.2 vs. 3.8%). In this sense, this study suggests the need to revise the systematic advice of prophylactic iron supplementation, particularly in nonanemic pregnant women, in order to avoid the risk of high Hb level and defends a more personalised iron supplementation according to the iron status during antenatal check-ups [30].

As far as we are aware, the study by Xu et al., 2020 [19], is the only study that explored the effect of high Hb concentration and pointed out that women with preconception Hb ≥150 g/L also had an increased risk of miscarriage [19].

However, the mechanisms underpinning this association remain to be determined. Raised Hb levels during pregnancy resulting from hypovolemia or haemoconcentration are frequently found in pre-eclampsia or pregnancy-induced hypertension [31,32]. Since both disorders induced by haemoconcentration seem to be associated with increased risk of stillbirth and perinatal mortality [32,33], similar pathological mechanisms may be involved in miscarriage [31,34]. In addition, haemoconcentration may also be due to iron excess in the organism [35,36]. The mechanism is not entirely clear, but haemoconcentration by increasing blood viscosity may impede the uteroplacental circulation, causing placental infarction, growth retardation, and ultimately foetal death [32].

On the other hand, the increased incidence of miscarriage has been associated with other maternal characteristics, such as advanced age or pre-pregnancy BMI ≥25 kg/m^2^ [37,38]. Once confirmed by our study, we considered them as potential confounding factors, and we found out that adjustments for age and BMI did, even using stratified analyses, not alter the association of Hb levels with miscarriages. This suggests that low and high Hb concentrations in the first trimester can predict the risk of miscarriage beyond well-established risk factors.

The main strength of our study is the large sample size, where all pregnant women attended by the ASSIR service of the Catalan Health Institute (about 70% of pregnant women in Catalonia) were included; therefore, the results are representative of our society. In addition, subgroup analyses and adjustments for potential confounders showed similar results, which suggest the robustness of our results. Additionally, we also analysed Hb concentrations continuously, using a restricted cubic spline transformation to account for non-linear effects, avoiding the undesirable property of assigning arbitrary categorizations; this approach was seldom performed in previous studies.

Some limitations should also be taken into account. The frequency of miscarriage in the current study was relatively low (5.5%). Most miscarriages occur in the first few weeks of pregnancy and a certain proportion does not result in medical intervention. It is likely that many early losses were unrecognized and, thus, not reported, which could lead to underestimation of the miscarriage frequency. Besides, our study involved young and healthy pregnant women living in the Mediterranean region, which might decrease the occurrence of miscarriage. Unfortunately, information on other haematological parameters, such as ferritin levels, was not available; therefore, we could not distinguish which specific type of anaemia or high Hb level is associated with miscarriage. Furthermore, information regarding pregnancy complications (e.g., preeclampsia or pregnancy-induced hypertension), diet, or physical activity was lacking, so we could not adjust for these factors in the multivariable analysis. Finally, it should be underlined that the Hb fluctuations in early pregnancy may yield random biases of miss-classification which tends to attenuate risk estimates. Nevertheless, the vast majority (80%) of Hb determinations in our study were done in a fairly specific gestational period within 9 and 11 weeks of gestation, which could reduce the Hb fluctuations due to the gestational moment of its determination.

## 5. Conclusions

The current study indicates that anaemia and high Hb concentrations in early pregnancy are associated with an increased risk of developing miscarriage. The thresholds of 110 to 140 g/L of maternal Hb might represent a potential target to lower the risk of miscarriage. Further studies are warranted to validate our results.

## Figures and Tables

**Table 1:** General and obstetric characteristics of pregnant women by Hb concentrations up to 14 weeks of gestation.

				Maternal Hb Concentrations Categories, n (%) ^a^			
		*N*	Mean Hb ± SD	Anaemia <110 g/L *n* = 358 (3.8)	Normal Hb 110–140 g/L *n* = 8505 (90.0)	High Hb ≥140 g/L *n* = 590 (6.2)	*p*-Value
Hb concentration up to 14 weeks of gestation (g/L), mean ± SD		9453	126.3 ± 9.1	103.0 ± 6.3 †	126.1 ± 6.8	143.2 ± 3.3 0 #	<0.001
Miscarriage, n (%)		520		30 (8.4) †	430 (5.1)	60 (10.2) #	<0.001
Age of mother at conception (years), mean ± SD		9453	29.9 ± 5.5	29.8 ± 6.2	29.9 ± 5.5	30.2 ± 5.4	0.51
	<20, n (%)	331	125.3 ± 8.4	16 (4.7)	306 (3.8)	9 (1.6)	0.003
	20–24, n (%)	1357	126.1 ± 9.7	69 (20.4) †	1199 (14.9)	89 (15.8)	
	25–29, n (%)	2498	126.5 ± 8.9	84 (24.8)	2263 (28.2)	151 (26.7)	
	30–34, n (%)	2713	126.7 ± 8.8	87 (25.7)	2442 (30.4)	184 (32.6)	
	35–39, n (%)	1685	126.0 ± 9.2	62 (18.3)	1519 (18.9)	104 (18.4)	
	≥40, n (%)	337	125.9 ± 10.3 *	21 (6.2) †	289 (3.6)	27 (4.8)	
Weight in the first trimester (Kg)		9008	65.2 ± 12.5	64.6 ± 12.6	65.2 ± 12.4	66.7 ± 12.6 #	0.017
Missing, n		445		25	384	36	
BMI in the first trimester (Kg/m^2^), mean ± SD		8935	24.8 ± 4.5	24.3 ± 4.4	24.7 ± 4.5	25.3 ± 4.6 #	0.002
	<18.5, n (%)	249	124.6 ± 9.4	16 (4.9)	220 (2.7)	13 (1.3)	0.001
	18.5-24.9, n (%)	5127	125.9 ± 8.8	189 (57.3)	4663 (57.9)	275 (50.4)	
	≥25, n (%)	3559	127.0 ± 9.2 *	125 (37.9)	3176 (39.4)	258 (47.3) #	
	Missing, n	518		28	446	44	
Smoking habit, n (%)							
	No	6926	126.0 ± 9.2	283 (85.2)	6252 (79.6)	391 (74.5)	0.001
	Yes	1781	127.1 ± 8.7 *	49 (14.8) †	1598 (20.4)	134 (25.5) #	
	Missing, n			640	106	106	
Previous births (number), mean ± SD		9323	0.8 ± 0.9	1.1 ± 1.1 †	0.8 ± 0.9	0.7 ± 0.9 #	<0.001
	Missing, n	130		6	112	12	
Parity, n (%)							
	Nulliparous	4064	126.9 ± 8.9	122 (34.7)	3650 (43.5)	292 (50.5)	<0.001
	Parous	5259	125.8 ± 9.2 *	230 (65.3) †	4743 (56.5)	286 (49.5) #	
	Missing, n	130		6	112	12	
Pregnancies (number), mean ± SD		9323	2.2 ± 1.2	2.6 ± 1.5 †	2.2 ± 1.2	2.1 ± 1.2	<0.001
Missing, n		130		6	112	12	
Previous miscarriage, n (%)							
	0	6609	126.4 ± 8.9	223 (63.4)	5989 (71.4)	397 (68.7)	0.003
	1	2067	126.2 ± 9.5	90 (25.6)	1839 (21.9)	138 (23.8)	
	≥2	647	125.6 ± 9.8	39 (11.1) †	565 (6.7)	43 (7.4)	
	Missing, n	130		6	112	12	

Values are expressed in mean ± SD (standard deviation) or number (%). Abbreviations: BMI, body mass index. ^a^ Percentages based on non-missing values. * Statistically significant differences in maternal Hb concentrations for intragroup comparisons at *p <* 0.05 as derived from Student’s t/ANOVA tests, as appropriate. *p*-values for the differences between maternal Hb concentrations categories (anaemia, normal Hb, or high Hb concentrations) as derived from ANOVA or chi-square tests, as appropriate. *p* < 0.05 for the differences between † anaemia versus normal Hb concentrations and # high Hb versus normal Hb concentrations.

**Table 2:** Associations between maternal Hb concentrations up to 14 weeks of gestation and other maternal characteristics and risk of miscarriage.

			Miscarriage		Unadjusted Model		Adjusted Model *	
Maternal Characteristics		No. of Participants	*n*	%	OR (95% CI)	*p*-Value	OR (95% CI)	*p*-Value
Hb concentration up to 14 weeks of gestation (g/L)								
	<110	358	30	8.4	1.72 (1.17 to 2.52)	0.006	2.08 (1.35 to 3.20)	<0.001
	110–139	8505	430	5.1	1.00 (ref.)		1.00 (ref.)	
	≥140	590	60	10.2	2.13 (1.60 to 2.82)	<0.001	1.78 (1.25 to 2.54)	0.001
Maternal age at conception (years)								
	<20	331	17	5.1	1.28 (0.76 to 2.18)	0.35	1.10 (0.59 to 2.07)	0.75
	20–24	1357	56	4.1	1.02 (0.73 to 1.43)	0.90	0.80 (0.53 to 1.20)	0.29
	25–29	2498	101	4.0	1.00 (ref)		1.00 (ref)	
	30–34	2716	148	5.5	1.37 (1.06 to 1.77)	0.017	1.36 (1.01 to 1.84)	0.041
	34–39	1685	122	7.2	1.85 (1.41 to 1.77)	<0.001	2.01 (1.47 to 2.75)	<0.001
	≥40	337	52	15.4	4.33 (3.03 to 6.18)	<0.001	4.83 (3.22 to 7.25)	<0.001
BMI in the first trimester (Kg/m^2^)								
	<18.5	249	6	2.4	0.49 (0.22 to 1.11)	0.09	0.67 (0.29 to 1.54)	0.35
	18.5–24.9	5127	246	4.8	1.00 (ref)		1.00 (ref)	
	≥25	3559	217	6.1	1.29 (1.07 to 1.55)	0.008	1.17 (0.94 to 1.45)	0.154
Smoking habit								
	No	6926	340	4.9	1.00 (ref)		1.00 (ref)	
	Yes	1781	74	4.2	0.84 (0.65 to 1.09)	0.18	0.84 (0.63 to 1.10)	0.20
Parity								
	Nulliparous	4064	247	6.1	1.00 (ref)		1.00 (ref)	
	Parous	5259	261	4.9	0.81 (0.67 to 0.96)	0.021	0.69 (0.55 to 0.86)	0.001
Previous miscarriage								
	0	6609	354	5.4	1.00 (ref)		1.00 (ref)	
	1	2067	111	5.4	1.00 (0.81 to 1.24)	0.98	0.87 (0.65 to 1.64)	0.25
	≥2	647	43	6.7	1.26 (0.91 to 1.74)	0.16	1.11 (0.75 to 1.64)	0.61

BMI, body mass index; OR, odds ratio; CI, confidence interval. * Logistic regression model was mutually adjusted for all characteristics displayed in this table.

**Table 3:** Sensitivity analyses. Associations between maternal Hb concentrations up to 14 weeks of gestation and risk of miscarriage.

			Miscarriage		Adjusted Model *	
Maternal Characteristics		No. of Participants	*n*	%	OR (95% CI)	*p*-Value
Only cases documented up to 14 weeks of gestation †		9366	433	4.6		
	Hb concentrations (g/L)					
	<110	353	25	7.1	2.11 (1.32 to 3.36)	0.002
	110–139	8434	359	4.3	1.00 (ref.)	
	≥140	579	49	8.5	1.66 (1.12 to 2.47)	0.012
Only cases documented between 15 and 24 weeks of gestation ‡		9020	87	1.0		
	Hb concentrations (g/L)					
	<110	333	5	1.5	1.93 (0.69 to 5.41)	0.21
	110–139	8146	71	0.9	1.00 (ref.)	
	≥140	541	11	2.0	2.38 (1.12 to 5.10)	0.025
Excluding cases documented within 11 and 14 weeks of gestation with a period of fewer than 2 weeks from the date of the Hb tests §		9299	366	3.9		
	Hb concentrations (g/L)					
	<110	347	19	5.5	1.95 (1.15 to 3.32)	0.013
	110–139	8374	299	3.6	1.00 (ref.)	
	≥140	578	48	8.3	2.20 (1.48 to 3.27)	<0.001
All pregnancy loss documented up to and beyond 24 weeks of gestation ¶		9488	555	5.9		
	Hb concentrations (g/L)					
	<110	362	34	9.4	2.24 (1.50 to 3.36)	<0.001
	110–139	8536	461	5.4	1.00 (ref.)	
	≥140	590	60	10.2	1.66 (1.17 to 2.37)	0.005

BMI, body mass index; OR, odds ratio; CI, confidence interval. * Logistic regression model was adjusted for the same characteristics or covariates as Table 2. † Of the 520 miscarriages, 87 were excluded. ‡ Of the 520 miscarriages, 433 were excluded. § Of the 520 miscarriages, 154 were excluded. ¶ Foetal deaths were defined as the sum of miscarriages and stillbirths. Stillbirths were defined as deliveries of at least 24 weeks of gestation that, at any time after delivery, the foetus did not show any signs of life.

**Table 4:** The associations of haemoglobin levels up to 14 weeks of gestation with the risks of miscarriage stratified by BMI in the first trimester and maternal age at conception.

			Miscarriage		Adjusted Model *		
Maternal Characteristics		No. of Participants	*n*	%	OR (95% CI)	*p*-Value	*p* for Interaction
BMI <25 Kg/m^2^		5376	252	4.7			0.13
	Haemoglobin level (g/L)						
	<110	205	20	9.8	2.77 (1.66 to 4.62)	<0.001	
	110–139	4883	207	4.2	1.00 (ref.)		
	≥140	288	25	8.7	2.25 (1.40 to 3.61)	0.001	
BMI ≥25 Kg/m^2^		3559	217	6.1			
	Haemoglobin level (g/L)						
	<110	125	8	6.4	1.33 (0.63 to 2.78)	0.45	
	110–139	3176	186	5.9	1.00 (ref.)		
	≥140	258	23	8.9	1.45 (0.88 to 2.39)	0.14	
Maternal age ≤ 29 years		4187	175	4.2			0.55
	Haemoglobin level (g/L)						
	<110	169	11	6.5	2.14 (1.05 to 4.34)	0.036	
	110–139	3768	142	3.8	1.00 (ref.)		
	≥140	250	22	8.8	2.40 (1.47 to 4.24)	0.002	
Maternal age > 29 years		5266	345	6.6			
	Haemoglobin level (g/L)						
	<110	189	19	10.1	2.09 (1.24 to 3.52)	0.006	
	110–139	4737	288	6.1	1.00 (ref.)		
	≥140	340	38	11.2	1.56 (1.01 to 2.40)	0.044	

BMI, body mass index; OR, odds ratio; CI, confidence interval. * Logistic regression model was adjusted for maternal age (≤29, >29 years; for BMI analysis), BMI (<25, ≥25 Kg/m^2^; for maternal age analysis), parity (nulliparous, parous), smoking (no, yes), and prior miscarriage (0, 1, ≥2).

## Data Availability

Data available on request due to restrictions privacy. The data presented in this study are available on request from the corresponding author (victoria.arija@urv.cat).
